# Anxiety, Depression, and Satisfaction With Life Among College Students in China: Nine Months After Initiation of the Outbreak of COVID-19

**DOI:** 10.3389/fpsyt.2021.777190

**Published:** 2022-01-18

**Authors:** Pei Xiao, Liang Chen, Xiaoqin Dong, Zhiya Zhao, Jincong Yu, Dongming Wang, Wenzhen Li

**Affiliations:** ^1^Center for Non-communicable Disease Management, National Center for Children's Health, Beijing Children's Hospital, Capital Medical University, Beijing, China; ^2^Department of the Prevention and Treatment of Leprosy, Wuhan Institute of Dermatology and Venereology, Wuhan, China; ^3^Wuhan Emergency Medical Center, Wuhan, China; ^4^Department of Social Medicine and Health Management, School of Public Health, Tongji Medical College, Huazhong University of Science and Technology, Wuhan, China; ^5^Education and Counseling Center for Psychological Health, Zhongnan University of Economics and Law, Wuhan, China; ^6^Department of Occupational and Environmental Health, School of Public Health, Tongji Medical College, Huazhong University of Science and Technology, Wuhan, China; ^7^Key Laboratory of Environment and Health, Ministry of Education and Ministry of Environmental Protection, and State Key Laboratory of Environmental Health (Incubating), School of Public Health, Tongji Medical College, Huazhong University of Science and Technology, Wuhan, China

**Keywords:** depression, anxiety, adolescent psychology, epidemiology, satisfaction with life

## Abstract

**Background/Objective:**

Mental health problems are common among college students. This study sought to assess the prevalence and risk factors of depressive and anxiety symptoms and well-being among Chinese college students 9 months after initiation of the outbreak of COVID-19.

**Method:**

A cross-sectional study (*N* = 3,951, mean age = 19.58) was conducted from October to December 2020. An online survey was used to collect socio-demographic data, and the symptoms of depression and anxiety and satisfaction with life using Disorder 7-Item Scale (GAD-7), the Patient Health Questionnaire 9-Item Scale (PHQ-9), and the 5-items Satisfaction with Life Scale (SWLS).

**Results:**

The prevalence of depressive and anxiety symptoms was 59.35 and 54.34%, respectively, and the score of satisfaction with life was 20.51 ± 6.42 among Chinese college students during the pandemic. After controlling for covariates, students in urban areas (AOR = 0.73, 95% CI = 0.61–0.87), with good family economic levels (AOR = 0.77, 95% CI = 0.66–0.91), and having psychological counseling (AOR = 0.55, 95% CI = 0.42–0.73) were positively associated with depression symptoms; meanwhile, higher anxiety symptoms were observed among medical students (AOR = 0.81, 95% CI = 0.69–0.95). Besides, healthy lifestyle such as regular physical activity and diet was associated with depression and anxiety symptoms. Multiple linear models revealed that medical students (β = 0.479, *P* = 0.031), those with good family economic level by self-evaluation (β = 1.283, *P* < 0.001 for good; β = 3.013, *P* < 0.001 for general), good academic performance by self-evaluation (β = 1.786, *P* < 0.001 for good; β = 3.386, *P* < 0.001 for general), learning burden (β = 1.607, *P* < 0.001 for general; β = 2.117, *P* < 0.001 for light), regular physical activity (β = 0.859, *P* < 0.001), daily routine (β = 1.289, *P* < 0.001), diet (β = 1.714, *P* < 0.001), and sufficient sleep (β = 1.408, *P* < 0.001) had more score of SWLS (all β > 0, *P* < 0.05), while senior students (β = −1.053, *P*=0.009), students having psychological counseling (β = −1.753, *P* < 0.001), and drinking (β = −0.743, *P* = 0.012) had lower satisfaction with life.

**Conclusions:**

These findings suggest that more attention should be paid to psychological health among college students, especially during and after the COVID-19 outbreak. Policy makers and educators should help college students develop a healthy lifestyle with regular diet and exercise to promote the psychological health of college students.

## Introduction

The Coronavirus Disease 2019 (COVID-19) caused by SARS-CoV-2, was first reported in December 2019, and rapidly spread around the world (1). To control the pandemic, the stringent COVID-19 control measures were imposed including strict stay-at-home policy for all residents and postpone the start of school in China. The COVID-19 epidemic disrupted residents' normal life, sleep and eating patterns, and increased social isolation and disappointment in life, which threatened their psychological health. Psychological problems were caused by all kinds of negative news such as increasing number of patients and deaths, lack of medical resources, etc. (2). Studies reported that psychiatric symptoms including anxiety and depression among the public have remained even more serious during the SARS epidemic and after 1, 30 months, and longer (3). In China, students are required to study online at home and cannot return to school until September or October 2020, and previous studies indicated that the effects of the pandemic on students may linger for a period beyond the peak of the COVID-19 pandemic itself (4) and, thus, it is necessary to assess psychological situations.

Mental health among college students has been an increasing concern, and many studies have been conducted to assess the mental health among college students. There is no doubt that mental disorders among college students is widespread and common in China and other countries (5, 6), and demographic variables such as gender and lifestyles have been explored to be associated with metal health among college students (7–9). For instance, among female students, smoking and drinking were reported to be associated with higher rates of anxiety and depression among college students. The COVID-19 pandemic situation has brought them into renewed focus, a recent study conducted in Sichuan Province from April 2020 to May 2020 with 521 university students showed 19.0% of respondents reported distress, and 31.5, 8.1, and 5.8% of them reported mild, moderate, and severe anxiety, respectively, by using 20-item Self Reporting Questionnaire and Self-Rating Anxiety Scale (10). Online classes and class projects given the lack of in-person support from instructors or teaching assistants may increase class workload, which may further increase their mental pressure (11). To understand and evaluate the psychological features among college students back to school after the epidemic, we conducted a national cross-sectional study to comprehensively describe college students' psychological situations and satisfaction with life.

## Methods

### Study Design and Participants

A national cross-sectional study with multistage cluster random sampling method was conducted among college students from October to December 2020. We first divided China into three regions: eastern (11 provinces), central (8 provinces), and western (12 provinces) based on the geographical area (according to the China health and family planning statistical yearbook), and two provinces were selected from each region randomly by drawing lots. Second, within each province, two colleges were randomly selected in the provincial capital. If there were too few colleges in the capital city then another city would be added. Finally, 12 college schools were selected in the present study. The questionnaires were completed through an online survey platform (“SurveyStar,” Changsha Ranxing Science and Technology, Shanghai, China). Students from the 12 colleges were randomly invited to complete the questionnaire. The study was approved by the Research Ethics Committee in Tongji Medical College, Huazhong University of Science and Technology, Wuhan, China (2020) S164. All agreed participants provided informed consent electronically prior to registration. If they agreed and registered, then they could see the questionnaire, or else they could not see the questionnaire and end up in the survey. Each mobile phone or computer could only be used once, all valid questionnaires identified by a background system were automatically entered into a data file and checked by two independent researchers. Questionnaires that took <3 min to fill out were excluded, 4,102 college students were selected randomly and filled out the questionnaires, and 3,951 students completed the valid surveys with no missing information. Questionnaires included demographic information, the 7-item Generalized Anxiety Disorder Scale (GAD-7), the Patient Health Questionnaire-9 (PHQ-9), and the Satisfaction with Life Scale (SWLS). Demographic information including gender, ethnicity, school type, major, grade, place of origin, only child or not, family income, self-rated family economic conditions, smoking, drinking, study burden, etc.

### Measurements

The GAD-7, a practical self-report anxiety questionnaire, has been bothered by each of the 7 core symptoms of generalized anxiety disorder. Response options are “not at all,” “several days,” “more than half the days,” and “nearly every day,” scored as 0, 1, 2, and 3, respectively. Therefore, GAD-7 scores range from 0 to 21, with scores of ≥5, ≥10, and ≥15 representing mild, moderate, and severe anxiety symptom levels, respectively (12). In addition, those with a total score ≤ 4 were considered as presenting no anxiety symptoms in the present analysis. The GAD-7 scale had good factorial validity and reliability with Cronbach's alpha coefficients of 0.82–0.89 and the validity of scale in assessing anxiety in Chinese has been confirmed (12, 13).

The Patient Health Questionnaire-9 (PHQ-9) is a self-report measure used to assess the severity of depression with the total scores categorized as follows: minimal/no depression (0–4), mild depression (5–9), moderate depression (10–14), or severe depression (14–21). In addition, those with a total score ≤ 4 were considered as presenting no depression symptoms in the present analysis, and the Chinese version of PHQ-9 had satisfactory reliability (with Cronbach's alpha coefficients of 0.869) and extensive sensitivity and specificity (15).

The SWLS is designed around the idea that one must ask subjects for an overall judgment of their life to measure the concept of life satisfaction, and is one of the most frequently used tools to measure the global cognitive judgment of satisfaction with one's life, which is comprised of 5 items rated on a 7-point Likert scale ranging from 1 (strongly disagree) to 7 (strongly agree), with higher values representing greater satisfaction (16). The reliability coefficient (Cronbach's alpha) of the scale in different studies ranged from 0.80 to 0.92 (16–18).

The Cronbach's alpha coefficients of GAD-7, PHQ-9, and SWLS in the present study were 0.935, 0.869, and 0.896, respectively.

### Statistical Methods

The data were tested for normal distribution. Descriptive analysis was compiled to describe the general data, and frequencies and percentages were used for count data. The chi-square test was used to compare the data for differences between basic situation and mental health statuses among various demographics. Only multivariate logistic regression models were used to evaluate the association between different factors and anxiety, depression and satisfaction with life. The dependent variables were anxiety, depression, and satisfaction with life; covariates including age, gender, residence area, specialized subject, family monthly income, father's education background, mother's education background, average monthly living cost, family economic level by self-evaluation, academic year, academic performance, learning burden, psychological counseling, smoking status, alcohol consumption, regular physical activity, regular daily routine, regular diet, sufficient sleep, BMI, and lost weight in the past month. *P* values < 0.05 (two-tailed) indicated that a difference was statistically significant. All data analysis was performed using IBM SPSS Statistics for Windows (Version 23.0).

## Results

The demographic characteristics of participants and the differences in their anxiety and depression statuses 9 months after the epidemic are shown in Table 1. Among 3,951 college students, the average age was 19.58 (SD 1.67), 2,277 (57.63%) were female students, 2,367 (59.91%) came from rural areas, and 1,360 (34.42%) were only children in their families. Of them, 59.35% had depression and 54.34% had anxiety. Significant differences in the students' depression levels were found based on residence area, family economic level by self-evaluation, academic performance by self-evaluation, learning burden, whether they do psychological counseling and lost weight in the past month, regular physical activity, daily routine, diet, and sufficient sleep. Students who came from rural areas were more likely to suffer from depression than those from urban areas (61.30 vs. 56.44%). In addition, significant differences in the anxiety level of students were found based on specialized subjects, family economic level by self-evaluation, academic performance by self-evaluation, learning burden, whether they do psychological counseling and lost weight in the past month, regular physical activity, daily routine, diet, sufficient sleep, and if they smoke. Non-medical students were more likely to have anxiety than others (55.79 vs. 51.14%).

**Table 1:** The anxiety and depression of college students in facing the epidemic of COVID-19.

**Variables**	***N*** **(%)**	**Depression**	**χ2/t**	***P***	**Cramer's V**	**Anxiety**	**χ2/t**	***P***	**Cramer's V**
Total	3,951	2,345 (59.35)				2,147 (54.34)			
Age (years), mean (SD)	19.58 ± 1.67	19.58 ± 1.81				19.61 ± 1.79			
**Gender**									
Male	1,674 (42.37)	980 (58.54)	0.789	0.374	0.014	892 (53.29)	1.303	0.254	0.018
Female	2,277 (57.63)	1,365 (59.95)				1,255 (55.12)			
**Residence area**									
Rural	2,367 (59.91)	1,451 (61.30)	9.298	0.002	0.049	1,294 (54.67)	0.613	0.256	0.008
Urban	1,584 (40.09)	894 (56.44)				853 (53.85)			
**Specialized subject**									
Medical	1,232 (31.18)	707 (57.39)	2.867	0.09	0.027	630 (51.14)	7.408	0.006	0.043
Non-medical	2,719 (68.82)	1,638 (60.24)				1,517 (55.79)			
**Family monthly income ($)**									
0–155	509 (12.88)	314 (61.69)	3.479	0.481	0.03	272 (53.44)	3.048	0.55	0.028
156–312	649 (16.43)	381 (58.71)				356 (54.85)			
313–469	1,592 (40.29)	925 (58.1)				856 (53.77)			
470–783	676 (17.11)	415 (61.39)				386 (57.1)			
≥784	525 (13.29)	310 (59.05)				277 (52.76)			
**Father's education background**									
Primary schools and below	693 (17.54)	415 (59.88)	2.476	0.48	0.025	372 (53.68)	1.791	0.617	0.021
Junior high school	1,557 (39.41)	939 (60.31)				847 (54.4)			
High school or technical secondary school	913 (23.11)	522 (57.17)				485 (53.12)			
Junior college or above	788 (19.94)	469 (59.52)				443 (56.22)			
**Mother's education background**									
Primary schools and below	1,129 (28.58)	665 (58.9)	0.82	0.845	0.014	617 (54.65)	3.108	0.375	0.028
Junior high school	1,444 (36.55)	870 (60.25)				760 (52.63)			
High school or technical secondary school	784 (19.84)	463 (59.06)				436 (55.61)			
Junior college or above	594 (15.03)	347 (58.42)				334 (56.23)			
**Only child in family**									
Yes	1,360 (34.42)	804 (59.12)	0.047	0.828	0.003	759 (55.81)	1.802	0.18	0.021
No	2,591 (65.58)	1,541 (59.48)				1,388 (53.57)			
**Average monthly living cost ($)**									
≤ 156	1,319 (33.38)	791 (59.97)	5.564	0.135	0.038	703 (53.3)	6.924	0.074	0.042
157–235	1,652 (41.81)	949 (57.45)				878 (53.15)			
236–313	676 (17.11)	412 (60.95)				384 (56.8)			
≥314	304 (7.69)	193 (63.49)				182 (59.87)			
**Family economic level by self-evaluation**									
Good	248 (6.28)	137 (55.24)	27.21	<0.001	0.083	127 (51.21)	14.155	<0.001	0.06
General	2,225 (56.31)	1,253 (56.31)				1,160 (52.13)			
Bad	1,478 (37.41)	955 (64.61)				860 (58.19)			
**Academic year**									
Freshman	1,191 (30.14)	678 (56.93)	4.404	0.221	0.033	640 (53.74)	2.217	0.529	0.024
Sophomore	1,191 (30.14)	713 (59.87)				636 (53.4)			
Junior	927 (23.46)	564 (60.84)				523 (56.42)			
Senior	642 (16.25)	390 (60.75)				348 (54.21)			
**Academic performance**									
Good	843 (21.34)	485 (57.53)	28.78	<0.001	0.085	487 (57.77)	18.265	<0.001	0.068
General	2,578 (65.25)	1,489 (57.76)				1,339 (51.94)			
Bad	530 (13.41)	371 (70)				321 (60.57)			
**Learning burden**									
Heavy	1,499 (37.94)	1,013 (67.58)	68.301	<0.001	0.131	989 (65.98)	140.537	<0.001	0.189
General	2,291 (57.99)	1,249 (54.52)				1,100 (48.01)			
Light	161 (4.07)	83 (51.55)				58 (36.02)			
**Psychological counseling**									
Yes	298 (7.54)	219 (73.49)	26.703	<0.001	0.082	217 (72.82)	44.354	<0.001	0.106
No	3,653 (92.46)	2,126 (58.2)				1,930 (52.83)			
**Smoking status**									
Yes	220 (5.57)	141 (64.09)	2.169	0.141	0.023	135 (61.36)	4.631	0.031	0.034
No	3,731 (94.43)	2,204 (59.07)				2,012 (53.93)			
**Alcohol consumption**									
Yes	538 (13.62)	337 (62.64)	2.79	0.095	0.027	308 (57.25)	2.123	0.145	0.023
No	3,413 (86.38)	2,008 (58.83)				1,839 (53.88)			
**Regular physical activity**									
Yes	999 (25.28)	547 (54.75)	11.714	<0.001	0.054	499 (49.95)	10.389	0.001	0.051
No	2,952 (74.72)	1,798 (60.91)				1,648 (55.83)			
**Regular daily routine**									
Yes	2,069 (52.37)	1,059 (51.18)	120.115	<0.001	0.174	1,006 (48.62)	57.241	<0.001	0.12
No	1,882 (47.63)	1,286 (68.33)				1,141 (60.63)			
**Sufficient sleep**									
Yes	1,833 (46.39)	880 (48.01)	182.369	<0.001	0.215	799 (43.59)	159.287	<0.001	0.201
No	2,118 (53.61)	1,465 (69.17)				1,348 (63.64)			
**Regular diet**									
Yes	2,504 (63.38)	1,313 (52.44)	135.551	<0.001	0.185	1,233 (49.24)	71.658	<0.001	0.135
No	1,447 (36.62)	1,032 (71.32)				914 (63.17)			
**BMI**									
Low weight	919 (23.26)	559 (60.83)	2.971	0.396	0.027	510 (55.5)	1.235	0.745	0.018
Normal	2,396 (60.64)	1,421 (59.31)				1,288 (53.76)			
Overweight	391 (9.9)	218 (55.75)				211 (53.96)			
Obesity	245 (6.2)	147 (60)				138 (56.33)			
**Lose weight in the past month**									
Yes	1,294 (32.75)	825 (63.76)	15.467	<0.001	0.063	762 (58.89)	16.031	<0.001	0.064
No	2,657 (67.25)	1,520 (57.21)				1,385 (52.13)			

Table 2 displays the satisfaction with life within each of the sociodemographic groups. In addition, the average score of satisfaction with life was 20.51 (SD 6.42) and significant differences were observed in residence areas, specialized subject, family monthly income, father's and mother's education background, family economic level by self-evaluation, academic year, academic performance by self-evaluation, learning burden, smoking, drinking, whether they do psychological counseling, regular physical activity, daily routine, diet, and sufficient sleep. Specifically, urban students were more likely to have higher satisfaction with life than rural students (20.98 vs. 20.19), students who lived healthy lifestyles with regular physical activity (21.65 vs. 20.12), regular daily routine (22.01 vs. 18.86), regular diet (22.24 vs. 19.01), sufficient sleep (21.59 vs. 18.63), no smoking (20.61 vs. 18.76), and no drinking (20.65 vs. 19.64) had higher satisfaction with life.

**Table 2:** The satisfaction with life of college students in facing the epidemic of COVID-19.

**Variables**	**mean (SD)**	**t/F**	***P***	**Cohen's d/eta-square**
Total	20.51 ± 6.42			
Age (years), mean (SD)	19.58 ± 1.67			
**Gender**				
Male	20.68 ± 6.57	1.397	0.16	0.045
Female	20.39 ± 6.30			
**Residence area**				
Rural	20.19 ± 6.26	−3.775	<0.001	0.123
Urban	20.98 ± 6.62			
**Specialized subject**				
Medical	20.87 ± 6.51	2.372	0.018	0.081
Non-medical	20.35 ± 6.37			
**Family monthly income ($)**				
0–155	20.1 ± 6.54	3.119	0.014	0.003
156–312	20.14 ± 6.42			
313–469	20.88 ± 6.53			
470–783	20.12 ± 6.1			
≥784	20.73 ± 6.29			
**Father's education background**				
Primary schools and below	19.71 ± 6.21	7.215	<0.001	0.005
Junior high school	20.34 ± 6.3			
High school or technical secondary school	20.93 ± 6.52			
Junior college or above	21.06 ± 6.63			
**Mother's education background**				
Primary schools and below	20.03 ± 6.08	4.103	0.006	0.003
Junior high school	20.5 ± 6.39			
High school or technical secondary school	21.02 ± 6.74			
Junior college or above	20.75 ± 6.62			
**Only child in family**				
Yes	20.75 ± 6.67	1.701	0.089	0.057
No	20.38 ± 6.28			
**Average monthly living cost($)**				
≤ 156	20.13 ± 6.22	2.517	0.056	0.002
157–235	20.64 ± 6.35			
236–313	20.71 ± 6.69			
≥314	20.99 ± 6.95			
**Family economic level by self-evaluation**				
Good	23.42 ± 6.47	68.104	<0.001	0.033
General	21.07 ± 6.21			
Bad	19.18 ± 6.43			
**Academic year**				
Freshman	20.27 ± 6.26	3.279	0.02	0.002
Sophomore	20.98 ± 6.47			
Junior	20.25 ± 6.49			
Senior	20.45 ± 6.45			
**Academic performance**				
Good	22.25 ± 6.34	92.093	<0.001	0.045
General	20.55 ± 6.25			
Bad	17.53 ± 6.30			
**Learning burden**				
Heavy	19.11 ± 6.56	60.406	<0.001	0.030
General	21.32 ± 6.13			
Light	22.05 ± 6.78			
**Psychological counseling**				
Yes	18.68 ± 6.79	−4.856	<0.001	0.301
No	20.66 ± 6.34			
**Smoking status**				
Yes	18.76 ± 6.82	−3.926	<0.001	0.280
No	20.61 ± 6.38			
**Alcohol consumption**				
Yes	19.64 ± 6.51	−3.339	<0.001	0.157
No	20.65 ± 6.39			
**Regular physical activity**				
Yes	21.65 ± 6.56	−6.406	<0.001	0.238
No	20.12 ± 6.32			
**Regular daily routine**				
Yes	22.01 ± 6.31	−15.911	<0.001	
No	18.86 ± 6.12			0.567
**Regular diet**				
Yes	22.24 ± 6.32	−16.351	<0.001	
No	19.01 ± 6.12			0.519
**Sufficient sleep**				
Yes	21.59 ± 6.35	−14.358	<0.001	
No	18.63 ± 6.09			0.476
**BMI**				
Low weight	20.21 ± 6.34	1.105	0.346	
Normal	20.55 ± 6.42			<0.001
Overweight	20.82 ± 6.42			
Obesity	20.75 ± 6.68			
**Lose weight in the past month**				
Yes	20.36 ± 6.32	−1.021	0.311	
No	20.58 ± 6.47			0.034

Table 3 reveals the factors associated with anxiety and depression among college students. Multivariate logistic regression models showed that college students who were from urban areas (adjusted odds ratio, AOR = 0.73, 95% CI, 0.61–0.87), good family economic level by self-evaluation (AOR = 0.77, 95% CI, 0.66–0.91), good academic performance by self-evaluation (AOR = 0.76, 95% CI, 0.61–0.94), light learning burden (AOR = 0.56, 95% CI, 0.39–0.79), psychological counseling (AOR = 0.55, 95% CI, 0.42–0.73), regular physical activity (AOR = 0.85, 95% CI, 0.72–0.99), regular daily routine (AOR = 0.77, 95% CI, 0.66–0.90), regular diet (AOR = 0.56, 95% CI, 0.48–0.65), and sufficient sleep (AOR = 0.62, 95% CI, 0.53–0.72) were less likely to have depression. However, those who were Juniors or Seniors and lost weight in the past month showed higher risk of depression. Factors significantly associated with anxiety among college students included light learning burden (AOR = 0.29, 95% CI, 0.20–0.41), psychological counseling (AOR = 0.46, 95% CI, 0.35–0.60), regular physical activity (AOR = 0.82, 95% CI, 0.70–0.96), regular diet (AOR = 0.55, 95% CI, 0.47–0.64), and sufficient sleep (AOR = 0.74, 95% CI, 0.63–0.87) and those who lost weight in the past month showed higher risk of anxiety.

**Table 3:** Multivariate logistic regression of anxiety and depression.

**Variables**	**Depression**			**Anxiety**		
	**AOR**	**95% CI**	***P***	**AOR**	**95% CI**	***P***
Age	1.00	0.95–1.06	0.92	1.00	0.95–1.05	0.96
Female (ref. = male)	0.93	0.80–1.09	0.375	0.96	0.83–1.12	0.621
Residence area (ref. = Rural)	0.73	0.61–0.87	0.001	0.85	0.72–1.02	0.077
Specialized subject (ref. = non–medical)	0.87	0.74–1.02	0.087	0.81	0.69–0.95	0.008
**Family monthly income (ref**. **=** **0–155$)**						
156–312	0.90	0.70–1.16	0.433	1.12	0.87–1.44	0.366
313–469	0.92	0.72–1.17	0.485	1.05	0.83–1.33	0.706
470–783	1.02	0.78–1.33	0.874	1.21	0.93–1.57	0.154
≥784	1.03	0.77–1.37	0.84	1.07	0.80–1.41	0.658
**Father's education background (ref**. **=** **primary schools and below)**						
Junior high school	1.06	0.86–1.29	0.588	1.09	0.89–1.32	0.406
High school or technical secondary school	0.98	0.77–1.24	0.847	1.04	0.82–1.31	0.766
Junior college or above	1.19	0.90–1.58	0.223	1.22	0.92–1.60	0.164
**Mother's education background (ref**. **=** **primary schools and below)**						
Junior high school	1.13	0.94–1.35	0.185	0.91	0.76–1.08	0.271
High school or technical secondary school	1.18	0.93–1.50	0.166	1.00	0.79–1.26	0.995
Junior college or above	1.07	0.80–1.43	0.657	0.92	0.69–1.22	0.548
Only child in family (ref. = no)	1.04	0.88–1.22	0.654	1.11	0.94–1.30	0.212
**Average monthly living cost (ref**. **=** **≤156$)**						
157–235	1.03	0.87–1.21	0.77	1.09	0.93–1.29	0.297
236–313	1.17	0.93–1.48	0.172	1.23	0.98–1.54	0.078
≥314	1.26	0.92–1.73	0.149	1.31	0.96–1.79	0.084
**Family economic level by self–evaluation (ref**. **=** **bad)**						
Good	0.77	0.66–0.91	0.002	0.86	0.73–1.01	0.059
General	0.77	0.56–1.07	0.121	0.76	0.55–1.06	0.102
**Academic year (ref**. **=** **Freshman)**						
Sophomore	1.20	1.00–1.44	0.052	1.02	0.86–1.23	0.79
Junior	1.29	1.04–1.61	0.023	1.16	0.94–1.44	0.174
Senior	1.49	1.12–1.99	0.007	1.19	0.89–1.57	0.24
**Academic performance (ref**. **=** **Bad)**						
Good	0.76	0.61–0.94	0.012	0.89	0.72–1.09	0.266
General	0.72	0.56–0.93	0.011	1.10	0.87–1.40	0.432
**Learning burden (ref**. **=** **Heavy)**						
General	0.65	0.56–0.75	<0.001	0.52	0.45–0.60	<0.001
Light	0.56	0.39–0.79	0.001	0.29	0.20–0.41	<0.001
**Psychological counseling (ref**. **=** **No)**	0.55	0.42–0.73	<0.001	0.46	0.35–0.60	<0.001
**Smoking status (ref**. **=** **No)**	0.91	0.66–1.26	0.575	1.15	0.84–1.58	0.377
**Alcohol consumption (ref**. **=** **No)**	1.08	0.87–1.34	0.467	1.03	0.83–1.27	0.795
**Regular Physical activity (ref**. **=** **No)**	0.85	0.72–0.99	0.042	0.82	0.70–0.96	0.016
**Regular daily routine (ref**. **=** **No)**	0.77	0.66–0.90	0.001	0.92	0.79–1.08	0.31
**Regular diet (ref**. **=** **No)**	0.56	0.48–0.65	<0.001	0.55	0.47–0.64	<0.001
**Sufficient sleep (ref**. **=** **No)**	0.62	0.53–0.72	<0.001	0.74	0.63–0.87	<0.001
**BMI (ref**. **=** **Low weight)**						
Normal	0.91	0.77–1.08	0.299	0.90	0.76–1.06	0.19
Overweight	0.80	0.61–1.04	0.099	0.95	0.73–1.24	0.724
Obesity	0.94	0.69–1.28	0.692	1.04	0.77–1.41	0.793
Lose weight in the past month (ref. = No)	1.33	1.14–1.54	<0.001	1.26	1.09–1.46	0.002

Multiple linear regression analysis (Table 4) presented that medical students and those with good family economic levels by self-evaluation, good academic performance by self-evaluation, learning burden, regular physical activity, daily routine, diet, and sufficient sleep had more score of SWLS (all of β > 0, *P* < 0.05), while senior students and students with psychological counseling and drinking had lower satisfaction with life (all of β < 0, *P* < 0.05).

**Table 4:** Multiple linear regression of satisfaction with life of college students.

**Variables**	**β**	**SE**	**t**	***P***
Age	0.090	0.076	1.174	0.24
Female (ref. = male)	−0.287	0.214	−1.344	0.179
Residence area (ref. = Rural)	0.143	0.248	0.578	0.563
Specialized subject (ref. = non–medical)	0.479	0.221	2.164	0.031
**Family monthly income (ref**. **=** **0–155$)**				
156–312	−0.153	0.351	−0.435	0.663
313–469	−0.108	0.335	−0.323	0.747
470–783	−0.374	0.367	−1.018	0.309
≥784	−0.369	0.398	−0.926	0.355
**Father's education background (ref**. **=** **primary schools and below)**				
Junior high school	0.305	0.279	1.095	0.274
High school or technical secondary school	0.566	0.332	1.708	0.088
Junior college or above	0.497	0.388	1.282	0.2
**Mother's education background (ref**. **=** **primary schools and below)**				
Junior high school	0.111	0.249	0.447	0.655
High school or technical secondary school	0.052	0.331	0.157	0.875
Junior college or above	−0.448	0.402	−1.112	0.266
Only child in family (ref. = no)	0.040	0.228	0.176	0.86
**Average monthly living cost (ref**. **=** **≤156$)**				
157–235	−0.001	0.233	−0.002	0.998
236–313	0.009	0.317	0.027	0.978
≥314	0.069	0.433	0.159	0.873
**Family economic level by self-evaluation (ref**. **=** **bad)**				
Good	1.283	0.226	5.678	<0.001
General	3.013	0.457	6.594	<0.001
**Academic year (ref**. **=** **Freshman)**				
Sophomore	0.359	0.255	1.406	0.16
Junior	−0.492	0.305	−1.616	0.106
Senior	−1.053	0.4	−2.633	0.009
**Academic performance (ref**. **=** **Bad)**				
Good	1.786	0.289	6.183	<0.001
General	3.386	0.336	10.084	<0.001
**Learning burden (ref**. **=** **Heavy)**				
General	1.607	0.202	7.975	<0.001
Light	2.117	0.493	4.293	<0.001
**Psychological counseling** **(ref**. **=** **No)**	−1.753	0.358	-4.891	<0.001
**Smoking status (ref**. **=** **No)**	−0.821	0.436	−1.883	0.06
**Alcohol consumption (ref**. **=** **No)**	−0.743	0.295	−2.517	0.012
**Regular Physical activity** **(ref**. **=** **No)**	0.859	0.226	3.799	<0.001
**Regular daily routine (ref**. **=** **No)**	1.289	0.222	5.805	<0.001
**Regular diet (ref**. **=** **No)**	1.714	0.213	8.05	<0.001
**Sufficient sleep (ref**. **=** **No)**	1.408	0.22	6.387	<0.001
**BMI (ref**. **=** **Low weight)**				
Normal	0.156	0.233	0.669	0.504
Overweight	0.138	0.372	0.371	0.71
Obesity	0.463	0.427	1.086	0.278
Lose weight in the past month (ref. = No)	−0.205	0.208	−0.983	0.326

## Discussion

College students considered vulnerable to psychological health are facing unprecedented levels of distress, especially during the COVID-19 pandemic. Due to the COVID-19 outbreak, schools in China have been locked down at all levels, and educational authorities have developed online portals and applications to deliver lectures and teaching activities, the uncertainty of academic development would have adverse impact on students' psychological health. Besides, students were required to report their daily health conditions and comply with prevention promotion of the COVID-19 and daily updates about surveillance and active cases (19), which may result in psychological distress. The high prevalence of mental problems is a warning that we should not ignore, particularly among college students although the COVID-19 situation has been better.

The findings of this study bring into focus the mental health and well-being of this specific population. Thus, far, most studies have been conducted within 1 or 2 months of the COVID-19 outbreak and focused on its immediate impact, and our study conducting online survey found that a majority of college students were experiencing depression and anxiety back to school after about 9 months of home isolation and study online. A nationwide cross-sectional survey was conducted among Chinese college students from February 4 to February 12, 2020 showed the prevalence of anxiety, depressive symptoms were 17.8 and 25.9% for college students (20), and another study conducted from March 8 to March 15, 2020 showed the prevalence was 27.1 and 39.2%, respectively (21), Luo et al. indicated that the pooled prevalence of depressive symptoms in Chinese university students was 26.0% during the COVID-19 pandemic (22), while Cao et al. reported the prevalence of anxiety was 24.9% (23). Our study showed a higher prevalence of anxiety (54.34%) and depressive (59.35%) symptoms simultaneously, the higher prevalence of anxiety and depression may be a reflection of overestimation by the tools, as the tools were different in these studies, such as the 21-item Depression, Anxiety, and Stress Scale (DASS-21). However, we still hold it may be more resulting from the residents being forced to quarantine at home and entertainment activities have been restricted for more than 9 months, and a study conducted among 1,242 Wuhan residents showed 27.5% had anxiety, 29.3% had depression (24), who were forced to isolate themselves in their homes, and all forms of gathering have been strictly prohibited. Besides, all kinds of news about the epidemic and deaths told through the Internet also aggravates their psychological burden. Furthermore, all college students had to study online by themselves, a learning burden made them more likely to be depressed and anxious. Moreover, our study suggested that the epidemic may have lasting effects on college students' psychological health, and the risk of psychological disorders may increase over time, which supported two recent studies which found negative impact of the pandemic on mental health may be continuous and long-term (25, 26). Therefore, effective interventions from policy makers, educators, and psychologists should be conducted among college students to provide timely and effective interventions for mental health benefits.

The present study showed regular and health lifestyle changes such as regular physical activity, daily routine, diet, and sufficient sleep were positively associated with mental health and satisfaction with life. As we all know, healthy lifestyle contributes to protect the population's mental health, and our study was consistent with previous studies, which showed a healthy lifestyle with a balanced diet and regular exercise were associated with lower levels of depression and anxiety symptoms among adolescents during COVID-19 (27). An unhealthy pattern has been found to worsen the mental state and cognitive functioning (28, 29), and numerous studies have reported that sustained adherence to healthy eating patterns can reduce markers of inflammation in humans (30), and heightened inflammation was linked to various mental health conditions, including mood disorders (31). In addition, regular physical activity is associated with lower levels of depression and anxiety symptoms, and the psychosocial mechanism hypothesis and behavioral mechanism hypothesis provided some explanation, and physical activity helps students for social interaction, self-efficacy, and perceived competence and improvements (32) as well as may improve self-regulation and coping skills which, in turn, helps students effectively stay positive mentally (33). Given the positive impact of healthy diet and lifestyle, it is expected that the combination of regular and good diet and regular physical activity would lead to greater benefits in the mental health and satisfaction with life than promoting one healthy lifestyle behavior alone during the COVID pandemic. Sleep problems could induce a poorer mental health status, and our study indicated that sufficient sleep was a protective factor for depression and anxiety, the possible mechanism was described in detail in a previous publication with individuals' sleep and mental health status interacting with each other (34). Juniors and seniors were more likely to have depression symptom, perhaps because they may experience greater pressure of study and employment than first-year students. Additionally, residence of family of origin was associated with depression, urban residents had fewer symptoms of depression, which may be related to their richer and more convenient learning resources, which may contribute to buffer their pressure.

The 5-item SWLS was used to measure subjective well-being. Our study showed the scores of the SWLS among college students during the COVID-19 pandemic was higher than it was during the severe acute respiratory syndrome (SARS) epidemic in 2003, which showed an average score with 19.45 among 381 college students (35). Faster and wider publicity including improvement in health literacy, the touching stories about the fight against the epidemic, and the spirit of unity and more social support may have improved the overall satisfaction of life among current college students compared with those in 2003. Besides, a wide variety of virtual meeting applications, video software, and apps such as WeChat, QQ, Tencent Conference, and Zoom were used frequently to connect to friends and family in COVID-19, which may be helpful for their well-being (11). Besides, the same as depression and anxiety symptoms, a healthy lifestyle and regular physical activity were advantages for improving satisfaction with life.

### Strengths and Limitations

Our findings have clinical and policy implications. First, psychology counselors in college should pay attention to the assessment of students' mental problems, communicating with their parents in a timely manner so as to implement effective intervention. In addition, health authorities and educators need to identify high-risk groups to conduct early psychological intervention. Moreover, healthy lifestyle behaviors (regular diet, physical activity, and sufficient sleep) should be promoted as an important preventive strategy to maintain their mental health. Some limitations in our study should be mentioned. First, the cross-sectional design precludes making causal inferences and no comparable pre-COVID-19 data were available within this study. Thus, longitudinal and retrospective studies are encouraged. Second, self-reported information may cause bias due to the social desirability effect and memory error. Thus, there is a need for better-designed studies with larger sample size and objective measure to provide more valuable information. Third, although the presence of mental problems was assessed by standardized questionnaires, these measures are not equivalent to clinical diagnoses, thus future studies with diagnostic interviews should be used. Fourth, we did not measure our participants about their degree of trauma in the outbreak, such as whether any their family members lost their jobs or even lives due to COVID-19, which might influence mental status. Fifth, although several confounders were adjusted in our study, there were still some other factors which were not included, such as a history of COIV-19 infection, admission to the hospital, vaccination, taking medication, and the number of online courses, which were related to COVID-19. Finally, the family's economic level was self-reported, and the assessment was biased and subjective.

## Conclusion

In conclusion, a higher prevalence of psychological symptom is found among college students especially for medical students after 9 months of COVID-19 outbreak. The findings of our study highlight the urgent need to develop interventions and preventive strategies to address the mental health of college students.

## Data Availability Statement

The original contributions presented in the study are included in the article/supplementary material, further inquiries can be directed to the corresponding author/s.

## Ethics Statement

The study was approved by the Research Ethics Committee in Tongji Medical College, Huazhong University of Science and Technology, Wuhan, China. The patients/participants provided their written informed consent to participate in this study.

## Author Contributions

WL, PX, LC, XD, and JY conceived and designed the study. ZZ, DW, JY, and WL participated in the acquisition of data. ZZ and WL analyzed the data. PX, LC, and XD drafted the manuscript. JY and DW revised the manuscript. All authors contributed to the article and approved the submitted version.

## Funding

The Fundamental Research Funds for the Central Universities (2019kfyXJJS032). The funder did not play any role in study design; in the collection, analysis, and interpretation of data; in the writing of the report; nor in the preparation, review, or approval of the manuscript.

## Conflict of Interest

The authors declare that the research was conducted in the absence of any commercial or financial relationships that could be construed as a potential conflict of interest.

## Publisher's Note

All claims expressed in this article are solely those of the authors and do not necessarily represent those of their affiliated organizations, or those of the publisher, the editors and the reviewers. Any product that may be evaluated in this article, or claim that may be made by its manufacturer, is not guaranteed or endorsed by the publisher.
